# *Quercus cerris* Leaf Functional Traits to Assess Urban Forest Health Status for Expeditious Analysis in a Mediterranean European Context

**DOI:** 10.3390/plants14020285

**Published:** 2025-01-20

**Authors:** Luca Quaranta, Piera Di Marzio, Paola Fortini

**Affiliations:** 1 Department Biosciences and Territory, University of Molise, 86090 Pesche, Italy; piera.dimarzio@unimol.it (P.D.M.); fortini@unimol.it (P.F.); 2 National Biodiversity Future Center (NBFC), 90133 Palermo, Italy

**Keywords:** Campobasso municipality, environmental data, phytosociological survey, plant functional traits, statistical analysis, plant stress, urbanization

## Abstract

In the Mediterranean basin, urban forests are widely recognized as essential landscape components, playing a key role in nature-based solutions by enhancing environmental quality and providing a range of ecosystem services. The selection of woody plant species for afforestation and reforestation should prioritize native species that align with the biogeographical and ecological characteristics of the planting sites. Among these, *Quercus cerris* L. (Turkey oak) is considered a promising candidate for urban reforestation. However, its fitness within urban forest environments remains poorly understood. This study aimed to identify suitable leaf functional traits for assessing the response of *Q. cerris* in urban forests and to analyze the main climatic variables influencing its performance in urban contexts. We also proposed practical, rapid monitoring tools to compare urban and natural forests across different seasons. The results demonstrated that *Q. cerris* experiences significant water stress in urban forests due to the combined effects of drought and high temperatures. To find the tools to mitigate this stress, the differences between leaf traits such as specific leaf area, thickness, and the contents of chlorophyll, anthocyanins, and flavonols in urban and natural forests were analyzed. Our findings underscore the high adaptability of *Q. cerris* to varied climatic and environmental conditions. This study provides a practical method for rapidly assessing the responses of tree species to climate change. In the future, this approach will be tested on other native species that are characteristic of Mediterranean forest ecosystems to help with choosing afforestation and reforestation strategies.

## 1. Introduction

Over the past fifty years, the gradual abandonment of farmland and grazing areas has facilitated the spontaneous regrowth of forests, particularly in Mediterranean Europe [1,2]. Despite these encouraging trends, the Mediterranean basin remains one of the most vulnerable regions globally to the impacts of climate change. Additionally, Mediterranean forests are significantly affected by high levels of degradation, which is primarily driven by anthropogenic activities [3]. These include the conversion of natural vegetation into extensive agricultural land, industrial development, and urban expansion [4]. The expansion of built-up areas replaces and transforms agricultural lands or natural forests, intensively impacting the environment and ecosystem functions. Large cities are the first to be affected; still, urbanization processes gradually affect smaller settlements and even remote rural villages [5]. The expansion of small cities generally proceeds slowly and determines a hybrid urban landscape, where portions of natural forests or abandoned lands are often incorporated into urban tissue. In this context, urban and peri-urban forests are increasingly recognized as critical components of the landscape, playing a pivotal role in nature-based solutions (NBSs). They contribute to environmental quality and multifunctionality by providing a wide range of ecosystem services, including regulating infiltration and stormwater runoff; mitigating microclimate extremes; reducing the urban heat island effect; combating soil, air, and water pollution; and limiting the spread of invasive species [6,7,8,9,10,11]. From the ecological perspective, urban and peri-urban forests are essential for maintaining the functions of the urban ecosystem [12,13,14,15,16].

Over the past fifty years, as most of the world’s population has transitioned to urban living, many researchers and international organizations have focused on understanding urban forests. They have examined important factors such as knowledge, management, socio-economic impact, and future scenarios, recognizing the importance of ecosystem services in urban environments and the vital role that cities play in the lives of people worldwide [17,18,19,20]. Less investigated, however, are the functional responses of tree species and the state of health of the entire urban forest ecosystem in the local context in Mediterranean cities, where plants may be affected by environmental stress as a consequence of climatic features such as air pollutants, altered solar radiation, lower wind speed, lower relative humidity, and higher rainfall. The last factor, however, does not translate into a higher soil water content due to the surface runoff caused by the high waterproofing of urban soils. The direct aspect of these effects is the increase in air temperature compared to the surrounding non-urbanized areas [21,22,23].

In Italy, to increase the resilience of urban areas, the national strategic plan for urban forestry compiled a list of woody plant species recommended for afforestation and reforestation (NRRP; Protection and enhancement of urban and extra-urban green—M2C4 3.1) [24] (https://www.nbfc.it (accessed on 30 November 2024)) [25]. This list encompasses the principal species constituting the flora of the Mediterranean biogeographic region within temperate forest ecosystems [26,27,28], and it includes both shrubs and tree species, with several oak species represented among the latter. Notably, *Quercus cerris* L. (Turkey oak) is identified as a key species for urban reforestation efforts. *Quercus cerris* (Sect. Cerris) is an autochthonous deciduous tree native to southern Europe and Asia Minor (chorotype: Euri-Mediterranean); at the European level, the species is mainly present in the Balkan and Italian peninsulas. This is a dominant species in the mixed forests of the Mediterranean basin covering the entire Italian territory (except Sardinia) for a total of around 280.000 ha of natural woods and those frequently occurring together with other oaks (*Quercus frainetto* Ten., *Q. petraea* (Matt.) Liebl., *Q. pubescens* Willd., *Q. robur* L.). The Turkey oak is found in urban areas at elevations from sea level up to the Apennines (1000–1200 m a.s.l.). It is highly adaptable to urban environments, making it an excellent choice for urban reforestation. As indicated in many papers, this species is particularly drought-tolerant and is suitable for planting as a single plant or in groups, in parks, gardens, squares, and tree-lined avenues, as well as in large and medium spaces [29]. Despite the growing attention to the role of trees in urban vegetation patches, the assessment of the ecological response of *Q. cerris* in urban forests at the local scale is still lacking.

One of the concepts that has been more utilized for identifying species’ environmental tolerance range values is the Ecological Indicator Value (EIV) system [30,31,32,33]. To date, the Ecological Indicator Values for Europe 1.0 (EIVE) [34] is the largest system database in Europe that can be used to retrieve information about specific species preferences in terms of light, temperature, soil moisture, soil nitrogen, and soil reaction, and it can be used to indirectly evaluate the ecological features of forests. Despite the robustness of the toll found in large applications in natural contexts [35,36,37,38], EIVE 1.0 is not suitable for indicating the real-time health status of trees in urban forests. This is particularly true because local conditions (reduced area, increased isolation, altered physical environment, etc.) can negatively impact the environmental tolerance of the species [4]. Indeed, the type of trees that can survive in a given location depends on their functional traits, which include all the physiological and morphological features that determine how they interact with and respond to their environment [39].

More suitable are the plant functional traits (PFTs), which are defined as the morphological, anatomical, physiological, biochemical, and phenological characteristics of plants that represent ecological strategies and determine how plants respond to environmental factors [40,41,42,43,44,45]. Numerous functional traits identified in the literature [40,46,47,48,49] can be utilized to evaluate the potential persistence and adaptive capacity of trees in urban forests facing various stressors. These stressors include climate change scenarios [50], heat islands, forest fragmentation, human impact, pollution, and competition from invasive species [51]. The functional traits documented to date are very numerous, encompassing various plant organs, including shoots, roots, and leaves. Since 2007, the TRY Plant Trait Database (https://www.try-db.org/ (accessed on 15 December 2024)) has aimed to collect a vast array of plant functional trait data from diverse sources, and it is highlighted as the most comprehensive archive of global plant data and is open access to the public [52]. Additionally, Pérez-Harguindeguy et al. [40] contributed to it by publishing a comprehensive framework for standardizing the measurement of plant functional traits worldwide. Among functional traits, those related to leaves are particularly advantageous due to their minimally invasive nature, suitability for studying plant communities, and repeatability over time, making them ideal for measuring characteristics of plants that influence their performance, survival, and ecological roles.

In this context, four leaf PFTs were selected for the purpose of the project: the specific leaf area (SLA), the leaf dry matter content (LDMC), the leaf thickness, and the chlorophyll content. This selection was based on their wide use in various studies and their ability to describe the strategies adopted by plants according to environmental conditions with non-invasive, repeatable, and expeditive analysis. In addition, two other pigments, i.e., anthocyanins and flavonols, were investigated on the basis of their relevant role in plant stress protection, but they have not been investigated thoroughly as traits [44,49,53,54,55,56,57,58].

The purposes of this paper are as follows:

1. To test and detect the selected leaf functional traits to study the response of tree species in urban forests;
2. To detect the main climatic variables that may affect urban forests;
3. To compare the health status of Quercus cerris in urban forests versus natural forests during different seasons;
4. To propose expedited and practical tools for monitoring the response of the trees in different urban forest conditions.

The results obtained in a Mediterranean urban context should help to monitor the health status of forest tree species in other climate regions, guiding the management of urban greenery, especially in large and medium-sized cities, which are experiencing increasingly rapid changes in environmental conditions.

## 2. Results

### 2.1. Community Description and Characterization

Based on the results of the coenological surveys (Table S1), it can be stated that the three stands were originally part of the natural forest, and these were separated over time due to the expansion of the city. Accordingly, these stands were classified within the association *Roso arvensis-Quercetum cerridis* Ubaldi 2003 (alliance *Crataego laevigatae-Quercion cerridis* Arrigoni 1997; order *Fagetalia sylvaticae* Pawlowski in Pawlowski, Sokolowski et Wallish 1928; class *Carpino-Fagetea* Jakucs ex Passarge 1968) [59].

*Quercus cerris* was found to be the dominant tree species across all of the sampled stands, alongside *Quercus frainetto*. Differences in the floristic composition were observed between natural forests and urban and peri-urban stands. The natural forest was characterized by high biodiversity, comprising 59 species, whereas the urban and peri-urban forests were deciduous mixed forests with a reduced floristic diversity of approximately 36 species, all of which were shared with the natural forest.

The tree layer in the natural forest predominantly consisted of *Quercus cerris*, *Q. petraea*, and *Q. frainetto*, while *Carpinus orientalis* dominated the shrub layer. Typical herbaceous forest species such as *Viola reichenbachiana*, *Fragaria vesca*, *Asperula taurina* subsp. *taurina*, and *Scutellaria columnae* were abundant. The tree layer in urban and peri-urban stands was primarily composed of *Quercus cerris*, *Q. frainetto*, and *Q. pubescens*, while the shrub layer included *Ligustrum vulgare*, *Prunus spinosa*, and *Ulmus minor*. The latter species was identified as the only indicator of anthropogenic influence within these forests.

**Figure 1:**
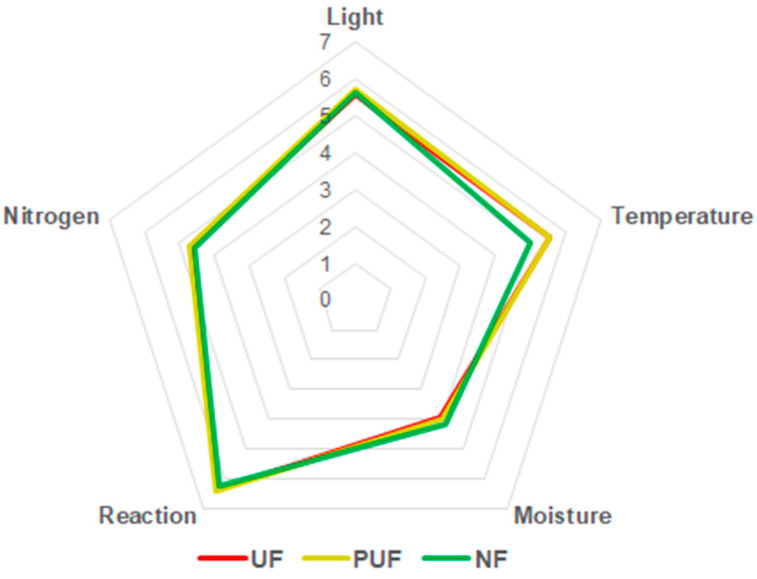
Kiviat diagram showing the weighted values of EIV 1.0 indexes (with an adimensional range value of 0 to 10) for the three stands (UF = urban forest; PUF = peri-urban forest; and NF = natural forest).

A Kiviat diagram was used to illustrate the Ecological Indicator Values (EIVs) [34] for each forest stand, providing insight into their responses to abiotic factors (Figure 1).

The weighted values were the following: L = 5.60, T = 5.53, M = 3.93, R = 6.36, and N = 4.67 for the UF stand; L = 5.74, T = 5.53, M = 3.97, R = 6.42, and N = 4.74 for the PUF stand; and L = 5.65, T = 5.00, M = 4.14, R = 6.26, and N = 4.58 for the NF stand. Comparing the EIVE indices of the three stands, no gradient was detected that passed from natural forest to urban forest, and the ecological requirements of the natural and urban forests appeared just marginally different.

### 2.2. Correlations Between Traits and Climatic Variables

**Figure 2:**
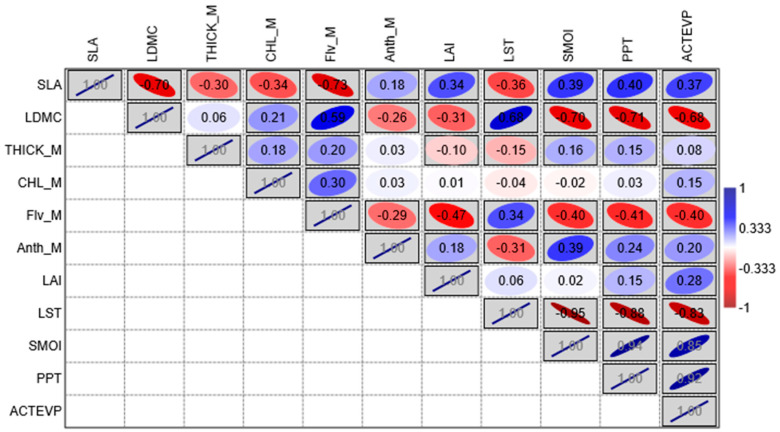
Correlation matrix showing the results of Pearson’s correlation analysis, which was performed on the entire dataset (11 variables; three stands; three time sampling). Pearson correlation coefficient values and directions are displayed with the following rule: positive correlation from white to blue and negative correlation from white to red on the color scale. Boxes are marked gray when *p* < 0.05. The inclination of the ellipse is representative of the positivity or negativity of the correlation, while its smaller or larger range indicates the intensity of the correlation. SLA = specific leaf area; LDMC = leaf dry matter content; THICK_M = leaf thickness; Flv_M = flavonol content; CHL_M = chlorophyll content; Anth_M = anthocyanin content; LAI = leaf area index; LST = land surface temperature; PPT = precipitation; SMOI = soil moisture; and ACTEVP = actual evapotranspiration.

The results of a Pearson’s correlation analysis, which was conducted on the entire dataset, are shown in Figure 2.

The specific leaf area (SLA) and leaf dry matter content (LDMC) diverged oppositely in terms of the variables, and they exhibited significant correlations with all the variables investigated. In detail, the SLA showed a moderate positive correlation with the climatic variables related to the presence of water in the stands (soil moisture—SMOI, precipitation—PPT, and actual evapotranspiration—ACTEVP) and a moderate negative correlation with the variable related to temperature (land surface temperature—LST). The correlation with anthocyanin content (Anth_M) and leaf area index (LAI) was weakly positive. Conversely, the leaf thickness (THICK_M), chlorophyll (CHL_M), and flavonol (Flv_M) content were moderately negatively correlated with the SLA. The relationships between LDMC and all the variables commented on above were opposite to those illustrated for SLA.

Interestingly, THICK_M showed a slightly positive correlation with all the pigments, and the correlation with Anth_M was not found to be statistically significant. Among the three pigments analyzed, CHL_M correlated the lowest with all the variables. Anth_M and Flv_M were negatively correlated with each other. They exhibited opposite correlations with SLA, LDMC, LST, and LAI variables. In contrast, the correlation with the variables related to the presence of water in the stands (PPT, ACTEVP, and SMOI) was negative for Flv_M and positive for Anth_M.

**Figure 3:**
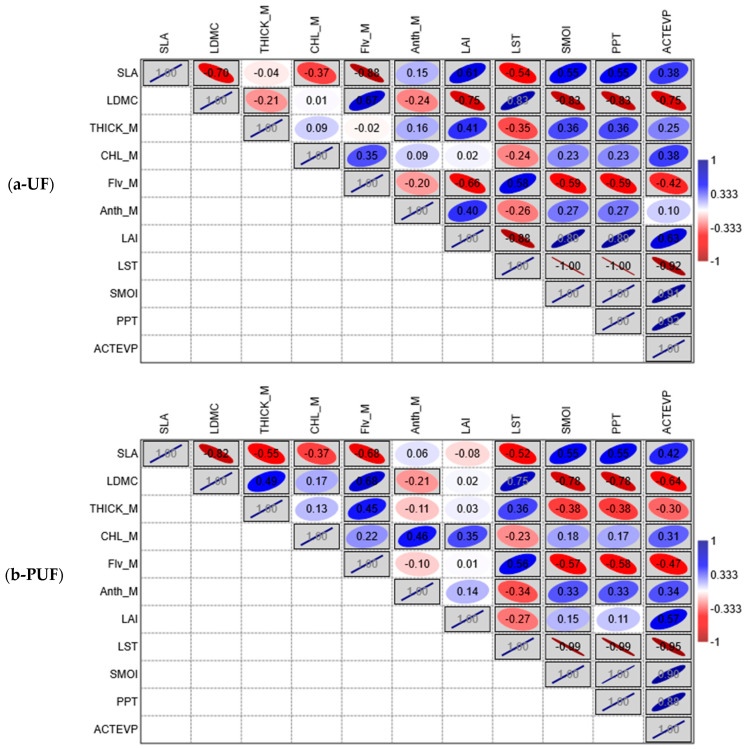

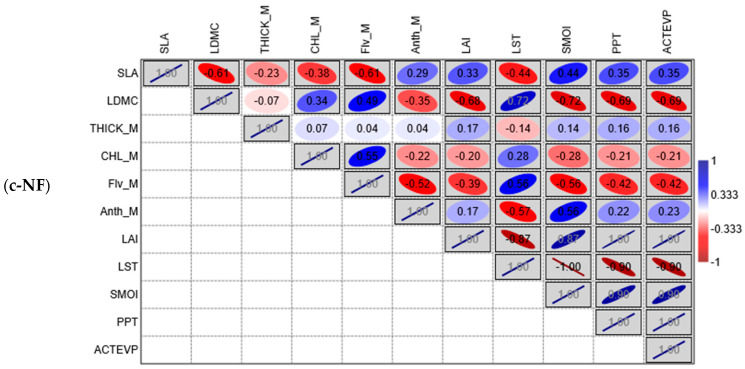
The correlation matrix shows the results of Pearson’s correlation analysis, which was performed separately on (**a**) UF—urban forest, (**b**) PUF—peri-urban forest, and (**c**) NF—natural forest stands. Pearson’s correlation coefficient values and directions are displayed with the following rule: positive correlation from white to blue and negative correlation from white to red on the color scale. Boxes are marked gray when *p* < 0.05. The inclination of the ellipse is representative of the positivity or negativity of the correlation, while its smaller or larger range indicates the intensity of the correlation. SLA = specific leaf area; LDMC = leaf dry matter content; THICK_M = leaf thickness; Flv_M = flavonol content; CHL_M = chlorophyll content; Anth_M = anthocyanin content; LAI = leaf area index; LST = land surface temperature; PPT = precipitation; SMOI = soil moisture; and ACTEVP = actual evapotranspiration.

Pearson’s correlation was performed on the three stands separately (Figure 3), and it highlighted a similar correlation, except for chlorophyll content, which presented a different correlation between the three sites. CHL_M in the urban forest negatively correlated with increasing temperature (LST), while the chlorophyll content in the natural forest positively correlated with temperature. On the other hand, variables related to soil water availability (PPT, SMOI, and ACTEVP) in urban and natural forests were oppositely correlated with chlorophyll.

**Figure 4:**
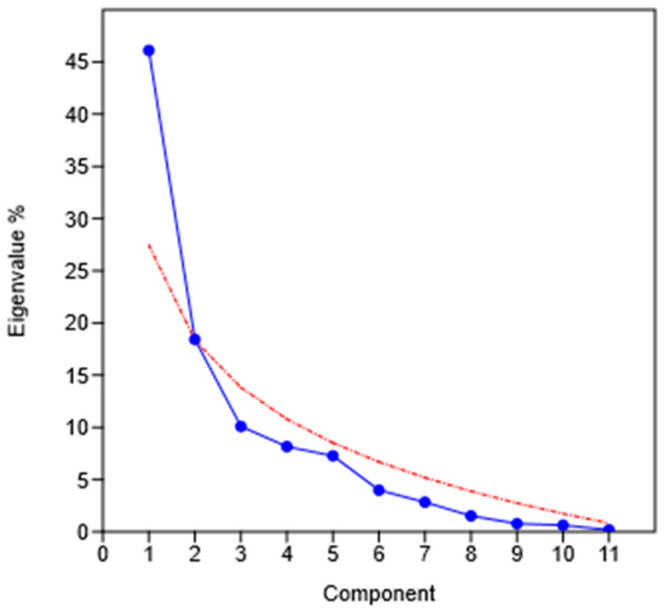
Principal component analysis scree plot of the eigenvalues (blue) with “broken stick” (red: eigenvalues expected under a random model), in which it is shown that the first two components explain more than 64% of the variance.

Furthermore, principal component analysis (PCA) was undertaken to evaluate the variation between the three forest stands in the three time-sampling periods (June, July, and September) and their relationships with leaf traits and climatic variables. The value of the Kaiser–Meyer–Olkin (KMO) measure was 0.76393, which is reported by PAST 4.17 instruction [60] as “good”, and the “scree plot” of the eigenvalues (Figure 4) showed that the higher part of the variability can be explained with the first axis.

**Figure 5:**
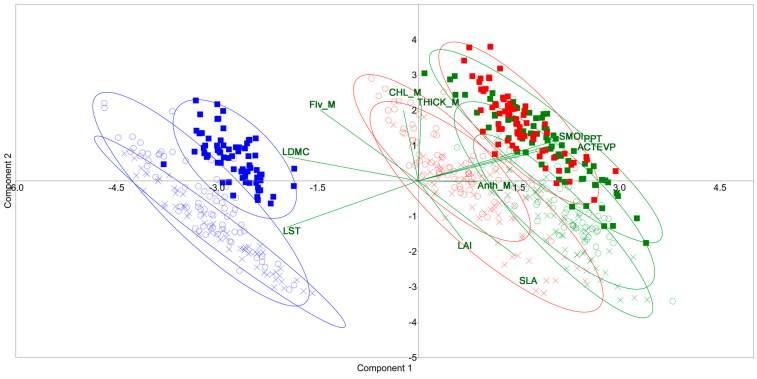
Scatter plot of the principal component analysis biplot depicting the relationship between the variables and the three stands. Urban forest (circle), peri-urban forest (X symbol), and natural forest (square) in three sampling times (June—green; July—red; September—blue). SLA = specific leaf area; LDMC = leaf dry matter content; THICK_M = leaf thickness; Flv_M = flavonol content; CHL_M = chlorophyll content; Anth_M = anthocyanin content; LAI = leaf area index; LST = land surface temperature; PPT = precipitation, SMOI = soil moisture; and ACTEVP = actual evapotranspiration.

Figure 5 shows the scatterplot of the component 1 and 2 scores, accounting for around 64% of the total variance. Along component one (with a variance of 46.10%), the data collected in June and July formed a distinct group from those harvested in September. These latter were located in the left part of the graph, and they were mainly associated with the variables LST, LDMC, and Flv_M; furthermore, the data related to urban and peri-urban forests overlapped, while those related to natural forests were separated and very close to each other. In the right part of the graph, the data collected in June and July were mainly associated with SMOI, PPT, and ACTEVP variables. The data of June show an overlap for the urban and peri-urban forests, while the natural forest data were slightly separated. For the data of July, the separation of the natural forest data was more evident from the other two forest stands. It was also evident that the data of June and July of the natural forest overlapped entirely, while the clouds of urban and peri-urban forest data shifted for the two sampling times.

### 2.3. Plant Functional Traits Variation in the Sampling Times

**Figure 6:**
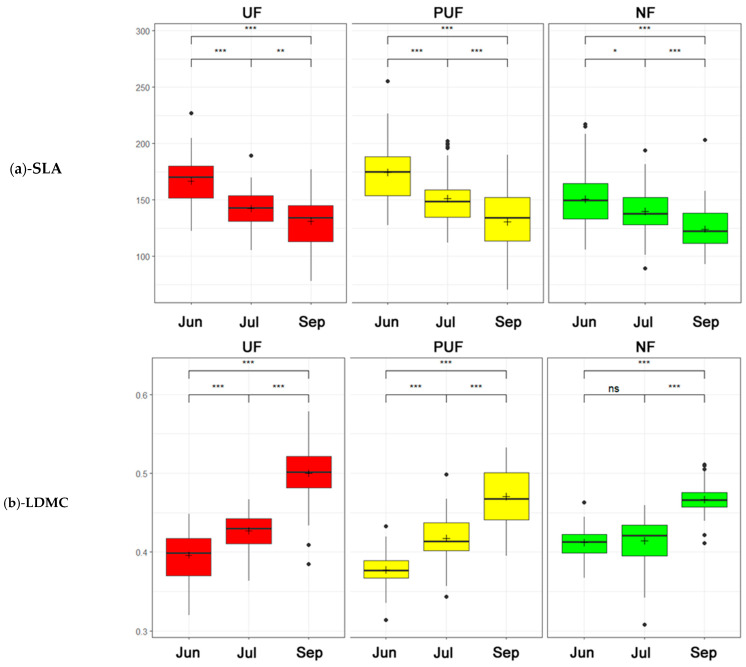
Two-way ANOVA box plots (a Tukey post hoc analysis was used to test statistical significance: ns = not significant, · = *p* < 0.1, * = *p* < 0.05, ** = *p* < 0.01, *** = *p* < 0.001) that describe the trends of the three stands (green—natural forest; yellow—peri-urban forest; and red—urban forest) in three periods (from left to right: Jun = June, Jul = July, and Sep = September). (**a**) Specific leaf area (SLA). (**b**) Leaf dry matter content (LDMC).

When considering the plant functional traits, the SLA values decreased in the three sampling times, with a higher statistically significant difference in the urban and peri-urban forests between June and July (Figure 6a). The LDMC values increased across all forest stands, though it was notable that the values observed in the natural forest did not reach a statistically significant difference when comparing June and July (Figure 6b).

**Figure 7:**
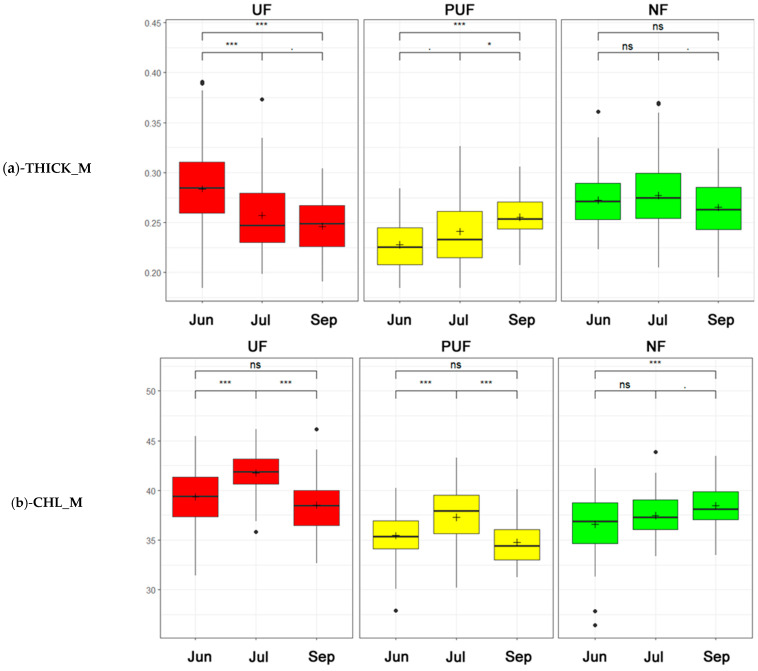
Two-way ANOVA box plots (a Tukey HSD post hoc analysis was used to test statistical significance: ns = not significant, · = *p* < 0.1, * = *p* < 0.05, *** = *p* < 0.001) that describe the trends of the three stands (green—natural forest; yellow—peri-urban forest; and red—urban forest) in three periods (from left to right: Jun = June, Jul = July, and Sep = September). (**a**) Leaf thickness (THICK_M). (**b**) Chlorophyll content (CHL_M).

THICK_M values exhibited three distinct trends: a decline in urban forest between June and July, an uptick in peri-urban forest, and a lack of significant variation globally in natural forest (Figure 7a). The CHL_M values in the urban and peri-urban forests were higher in July and overall higher in the urban forest. In contrast, the natural forest had constant growth in the three periods that became significant between the first and third periods (Figure 7b).

**Figure 8:**
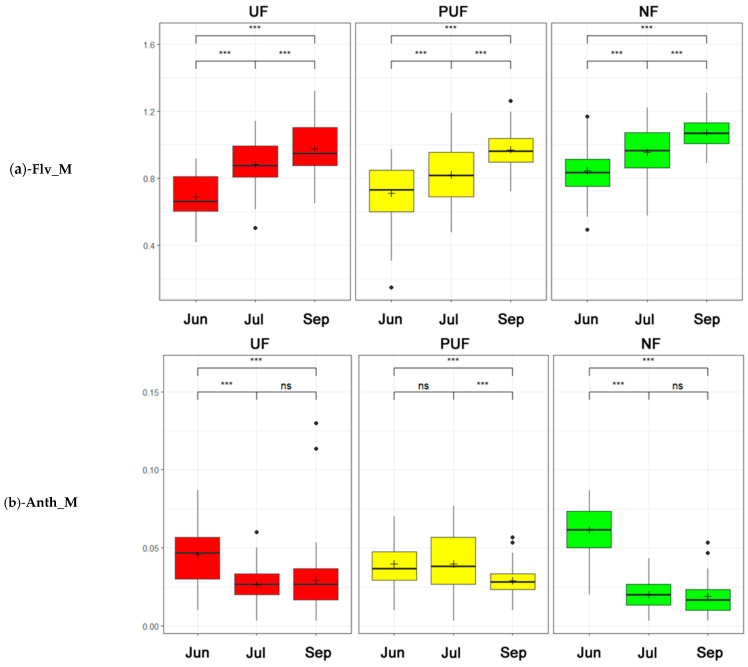
Two-way ANOVA box plots (a Tukey post hoc analysis was used to test statistical significance: ns = not significant, · = *p* < 0.1, *** = *p* < 0.001) that describe the trends of the three stands (green—natural forest; yellow—peri-urban forest; and red—urban forest) in three periods (from left to right: Jun = June, Jul = July, and Sep = September). (**a**) Flavonol content (Flv_M). (**b**) Anthocyanin content (Anth_M).

Flv_M values demonstrated considerable growth in all three stands and during the three sampling periods, with elevated values observed in the natural forest compared to the other two forests (Figure 8a). Anth_M values exhibited a comparable trend in natural and urban forests, with a decline that reached a stabilization in September. In contrast, the peri-urban forest demonstrated consistent stability throughout June and July, followed by a decrease in September (Figure 8b).

## 3. Discussion

The first finding from our phytosociological surveys testifies that the *Quercus cerris* urban and peri-urban forests were originally part of a continuum large natural forest, which underwent fragmentation due to the expansion of agricultural and built-up areas in the Campobasso municipality; furthermore, there was no evidence of planting or thinning in the three forests, and the impact of recreation in the urban forest was not significant. However, differences in the study area have inevitably occurred over time. The urban forest showed a poorer plant community composition, demonstrating the pressures of the city on nature. On the other side, the high presence of species shared with the natural forest (i.e., *Quercus cerris*, *Q. frainetto*, *Q. pubescens*, *Crataegus monogyna*, *Ligustrum vulgare*, *Prunus spinosa*, *Brachypodium sylvaticum*, and *Lathyrus venetus*) is a statement to nature’s resilience amidst urban development. These differences did not emerge when comparing the EIVE values among the three stand species; in fact, no evident gradient passing from the urban to the natural forest was detected. It would, therefore, seem outwardly that the ecological requirements differed only marginally. The similarity in the values provides that the three stands are effectively part of the same ecological typology of vegetation and that, consequently, the behavioral responses of the turkey oak in the three stands are mainly influenced by the urbanization gradient.

The leaf traits investigated (SLA, LDMC, leaf thickness, and chlorophyll content) were selected based on their relevance in describing the adaptive strategies of plant species and their use in many of the studies in the last few decades [46,47,48,58,61,62,63,64,65,66,67,68]. In addition, the contents of anthocyanins and flavonols were investigated due to their significant physiological role (i.e., photo-oxidative stress protection and high- and low-temperature protection). The selected variables were also related to the leaf area index and climatic variables (PPT, SMOI, ACTEVP, and LST) that heavily affect plant species development and growth [66]. Finally, *Quercus cerris* was monitored in the three stands (UF, PUF, and NF) and in three different periods (June, July, and September) to evaluate the trends of the variables considered.

We observed that the variables showed different meanings when comparing Pearson’s correlation results of the entire data set (three stands and three periods) with those obtained for the three stands separately (UF, PUF, and NF). The variables strongly correlated in the general analysis also had the same correlation in the analyses performed on the three subsets. In contrast, the ones that showed no significant correlation in the general analysis nullified each other with divergent correlations in the three stand analyses.

Amongst the correlations that showed the same trend in the three subsets, a strong negative correlation between SLA and LDMC (−0.70, see Figure 2) can be observed, which was quite predictable since these two indices described, on the one hand, opposite strategies for plants under different environmental conditions [46] and, on the other hand, they underwent variations according to leaf development (SLA decreasing and LDMC increasing as leaf age increases) [62,69,70,71]. These results were confirmed by the two-way ANOVA box plots calculated for each stand. *Q. cerris*, across the three forests, showed a decrease in specific leaf area (SLA) over the three periods, contrasting with an increase in LDMC (Figure 6). However, the variation of these trends differed between urban and natural forests. Specifically, the urban forest’s SLA values decreased more significantly than those in the natural forest. SLA is a critical trait that reflects a plant’s strategy for resource acquisition and utilization [72,73,74]. A less pronounced decrease in SLA suggests that NF plants maintain a relatively stable photosynthetic capacity under stress conditions [58]. Conversely, the significant increase in LDMC observed in the UF across all three periods suggested a strong adaptation to resource conservation and drought conditions [72,75,76]. This adaptive response, however, was absent in the natural forest during the first two periods (June and July) and only became evident in the last period (September).

The most interesting correlations were among SLA, LDMC, Flv_M, and Anth_M with the climatic variables. The results showed that an increase in temperature (LST) and a decrease in PPT, SMOI, and ACTEVP corresponded with a decrease in SLA values (generally increasing in developing leaves, in spring, and decreasing during leaf maturation), an increase in LDMC [62,69,70,71] (Figure 6), as well as an increase in temperature that corresponds with an increase in Flv_M and a decrease in Anth_M (Figure 2 and Figure 8).

The combination of these results showed that increasing temperatures and leaf development play a role in the regulation of the two pigments: Anth_M was present more in juvenile or senescent leaves and under low-temperature conditions [53,77,78,79,80], while Flv_M increased to enhance plant’s resilience to abiotic stressors such as high temperatures and drought [54]. The flavonol content in NF was significantly higher. Flavonols are crucial in protecting plants from environmental stress by acting as antioxidants and UV protectants.

**Figure 9:**
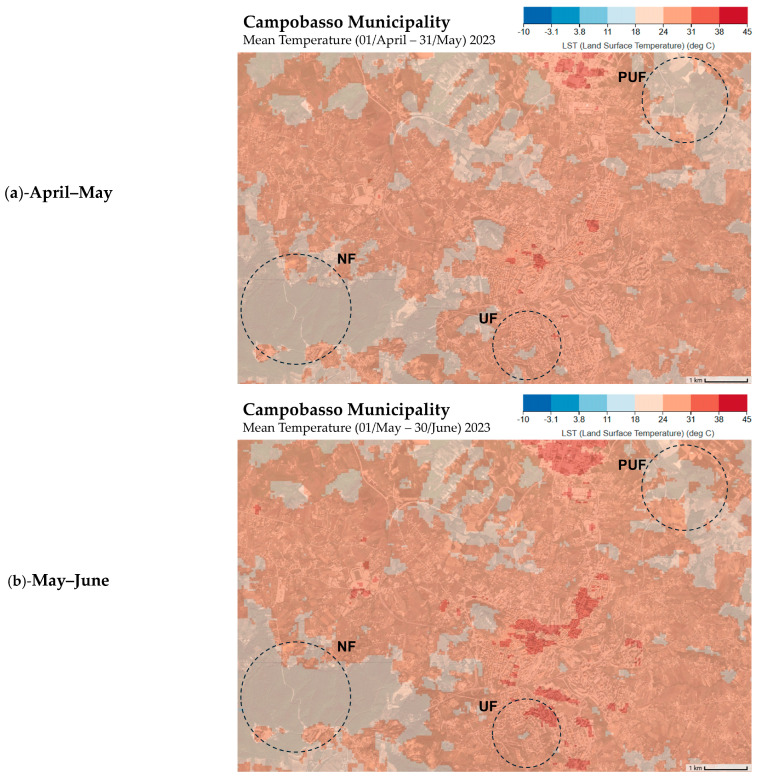

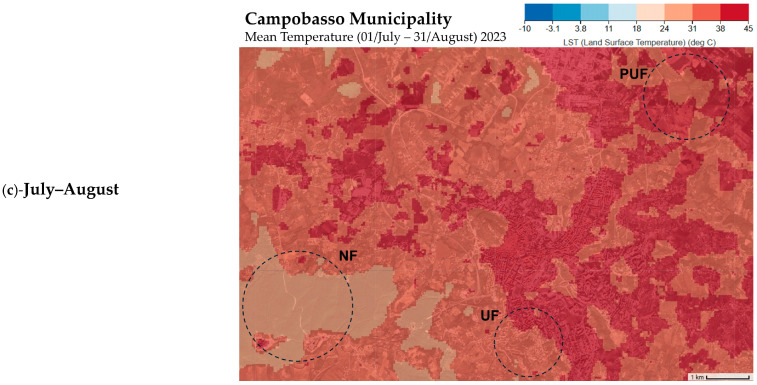
Land surface temperature (LST) maps of the Campobasso municipality (https://app.climateengine.org/climateEngine (accessed on 1 August 2024)), representing the mean temperature of the two months before each sampling date: (**a**) April–May for the June sampling; (**b**) May–June for the July sampling; and (**c**) July–August for the September sampling. NF—natural forest; PUF—peri-urban forest; and UF—urban forests.

The traits that showed few and weak correlations in the overall analysis were CHL_M and THICK_M. However, it is interesting to note that CHL_M showed a moderate negative correlation with SLA and a low positive correlation with LDMC (Figure 2). This result may be related to leaf development, which has a lower CHL_M content in young leaves (higher SLA) and increases as the leaf tissue matures (higher LDMC). Examining the results of the analyses performed on the three subsets, we observed that the divergent relationships of these two variables in the three stands may contribute to the weak correlation observed in the overall analysis. Specifically, CHL_M in urban and peri-urban forests was negatively correlated with LST and positively correlated with PPT, SMOI, and ACTEVP (Figure 3), whereas, in the natural forest, the correlation was the opposite. This discrepancy could be due to the significantly higher temperatures recorded, especially for the last sampling in September (mean temperature 1 July–31 August 2023) (Figure 9 and Table S2). Specifically, the UF (33.5 °C) and PUF (35.2 °C), compared to the NF (26.6 °C), exceeded the optimal threshold for chlorophyll synthesis (30 °C) [81,82].

In contrast, the positive correlation of CHL_M with climatic variables related to water availability emphasizes, in the urban and peri-urban forests, the water stress to which the two forests were subjected under synergetic conditions of drought and high temperatures. This correlation was expressed in the opposite way in the natural forest where, under near-optimal temperature conditions, *Q. cerris*, a species adapted to drought [83], did not appear to have any particular water requirements.

PCA confirmed the results obtained from the previous analysis. The environmental variables clearly drove the separation of the data collected in the three sampling times. LST was the most influencing climatic factor of the *Quercus cerris* responses registered in September, especially for urban and peri-urban stands; conversely, the variables related to water availability (PPT, ACTEVP, and SMOI) influenced the responses of the Turkey oak in June and July. This result is in accordance with those found by Salamanca-Fonseca et al. [84] around Bogotá city (Colombia), where they studied the effects of urban, peri-urban, and rural land covers on plant functional traits, confirming that urbanization locally increased temperatures and led to a reduction in evapotranspiration [85].

## 4. Materials and Methods

### 4.1. Study Area and Stands Selection

**Figure 10:**
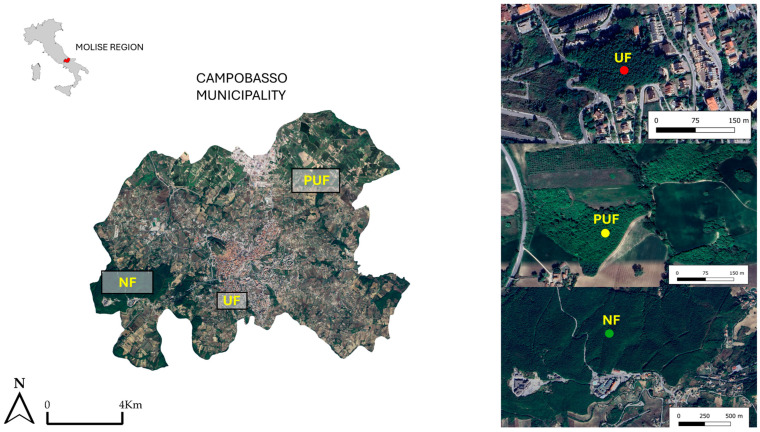
Location of the three stands, urban forest (UF), peri-urban forest (PUF), natural forest (NF), in the Campobasso municipality, and the location of the Molise region in Italy (red color).

The study area is located in the Campobasso municipality (Molise region, Southern Italy) (Figure 10), a small Mediterranean city that is 80 km from the Adriatic Sea; it is one of the eight Italian municipalities included in the studies of the National Biodiversity Future Centre (NBFC). The stands were chosen within a grid measuring cells of 1 km × 1 km developed by an interdisciplinary group (Spoke 5: Urban Biodiversity), who aimed to improve our knowledge of biodiversity in Italian cities and to provide new insights to protect and enhance nature in built-up areas (https://www.nbfc.it/en/environments (accessed on 1 August 2024)). The three cells were selected along a gradient of fragmentation and green cover. They corresponded to an urban forest (UF), a peri-urban forest (PUF), and a natural forest (NF) [50]. The natural forest is included in the Special Area of Conservation (SAC) IT7222295 “Monte Vairano” (Directive Habitat 92/43 EEC).

The macro-bioclimate of the three stands, according to Blasi et al. [86] and Rivas-Martínez et al. [26], can be defined as temperate oceanic/sub-Mediterranean when based on the classifications of the Ecoregions of Italy [28], and the study area falls under code 1C3a1. This code identifies the territory using the following characteristics: 1 = Temperate Division, C = Apennine Province, 3 = Southern Apennine Section, a = Campanian Apennine Subsection. The urban and natural forest stands are characterized by sandstones and conglomerates, and the peri-urban forest stands are characterized by feldspathic quartz sandstones. The altitudes vary from 587 to 875 m a.s.l. The average slope for the three stands is slight and around 10–15° (Table 1). Soil pH was measured with a portable field pH meter (YY-1033 soil pH meter, YIYEGO).

### 4.2. Sampling Protocol

The three studied stands were coppice forests converted into high forests of Turkey oak and Hungarian oak with a dominant layer covering around 80%. Before proceeding with the leaf measurements, the phytosociological surveys were done to describe the *Quercus cerris* community composition [87] and to obtain ecological information about the forests. The species and syntaxa nomenclature used followed Bartolucci et al. [88] and Biondi et al. [89], respectively.

The Ecological Indicator Values [34] (Table S3) were applied to the matrix of phytosociological relevés to calculate the weighted average response of each forest community to the abiotic factors, which express plant preferences for the following: temperatures, light, soil moisture, reaction, and nitrogen (using cover-abundance percentage values based on the Braun–Blanquet scale: r → 0.01%, + → 0.5%. 1 → 3%, 2 → 15%, 3 → 37.5%, 4 → 62.5%, 5 → 87.5%) [90].

Three sampling times were carried out in 2023 (June, July, and September), during three consecutive days under the same climatic conditions (no wind and no clouds), and in the 9:00–16:30 time frame—following Garnier et al. [64] and Vendramini et al. [61]—in order to monitor the trends of *Q. cerris* parameters as responses to environmental conditions. During the field sampling, the leaf area index (LAI) was measured using an AccuPAR LP-80 Ceptometer (METER group, Munich, Germany) (Table 2).

In each stand, a circle plot of one hectare (ha) area was identified, maintaining a 25 m buffer zone from the boundary of the forest. From the dominant layer (average height around 20 m), seven individuals were randomly sampled (at least 20 m apart) and ten leaves were collected for each one. For the leaf collection, different branches were cut in several exposures of the tree crown to represent the individual’s entire condition. The twigs were preserved in humidified air-tight bags for the subsequent steps [61].

The fresh weight (FW) was evaluated in the field with a portable balance (Kern-300 EMS) following the protocol proposed by Vendramini et al. [61]. The chlorophyll (CHL), anthocyanin (Anth), and flavonol (Flv) content were measured with the Opti-Sciences Inc. Multi-Pigment-Meter MPM-100. The thickness (THICK) was measured with a Neoteck Portable Digital Thickness Gauge. For better accuracy, these parameters were repeated in three different parts of the leaves: base, middle, and apex [91]. Subsequently, the three values were mediated (*_M). All the fresh leaves collected were scanned with an Epson GT15000 scanner (Epson Europe Electronics GmbH, Munich, Germany), and the images were analyzed with ImageJ 2.1.0/1.53c [92] to obtain the leaf area measurements. Finally, the leaves were placed in a dryer for 72 h at 70 degrees until there was complete water loss, and then the dry weight (DW) was measured.

The Plant Functional Traits, i.e., the specific leaf area (SLA) and leaf dry matter content (LDMC), were computed following Pérez-Harguindeguy et al. [40], Gottardini et al. [93], and Vendramini et al. [61] (Table 2).

### 4.3. Satellite Data

We used satellite data (Table 3) downloaded from the ClimateEngine.org website (https://www.climateengine.org/ (accessed on 1 August 2024)) to collect climatic data for the three different sampling periods: precipitation (PPT), soil moisture (SMOI), land surface temperature (LST), and actual evapotranspiration (ACTEVP). The data were averaged as the mean of the two months before each sampling period (April–May for the June sampling; May–June for the July sampling; and July–August for the September sampling).

### 4.4. Statistical Analysis

We collected field and laboratory data on *Quercus cerris* and organized all the information in a spreadsheet (Excel, 2016). The linear correlation between all the measured variables (traits and climatic variables) was calculated with Pearson’s r correlation coefficient using PAST 4.17 software [60] by performing an analysis on the entire dataset (11 variables; three stands; and three time-sampling periods). Subsequently, the same analysis was performed on the three stands (UF, PUF, and NF) separately. With respect to the strength of the correlation, the following scale was used: |r < 0.1| no correlation, |0.1 < r < 0.3| low correlation, |0.3 < r < 0.5| moderate correlation, |0.5 < r < 0.7| high correlation, and |0.7 < r < 0.1| very high correlation [95]. Subsequently, a principal component analysis (PCA) was performed using PAST 4.17 software. The data set was analyzed using the “correlation” option (which implies the normalization of the variables) because the variables were measured in different units. Finally, a two-way ANOVA (Rpackage stats version 4.2.1) [96] with a pairwise Tuckey HSD post hoc test (*p* ≤ 0.05) (Rpackage rstatix version 0.7.2) was used to evaluate the changes in the six traits (SLA, LDMC, THICK_M, CHL_M, Flv_M, and Anth_M) for the three stands (UF, PUF, and NF) during the three sampling periods.

## 5. Conclusions

Our study emphasizes the importance of leaf traits as indicators of the health status of *Quercus cerris* in both natural and urbanized areas. Additionally, we aimed to identify specific leaf traits suitable for rapid survey assessments to evaluate the stress levels experienced by this species in urban environments. *Quercus cerris* (Turkey oak) is a thermophilic species that is increasingly significant in the context of climate change due to its resilience to climatic extremes and drier conditions in natural habitats across Southern and Central Europe. Our findings highlight the strong adaptability of *Quercus cerris* to diverse climatic and environmental conditions. In urban contexts, we recommend long-term and seasonal monitoring of parameters such as anthocyanin and flavonol content. These traits have demonstrated their utility in detecting specific physiological adaptations to prevailing climatic conditions. They could serve as rapid indicators of *Quercus cerris* responses to climate change, helping to identify potential vulnerabilities and enabling more targeted monitoring strategies. Future work will aim to extend this protocol to other native species characteristic of Mediterranean forest communities that have already investigated by our research group from a phylogenetic point of view [97]. This approach will contribute to the development of practical tools for afforestation and reforestation efforts, particularly in urban and peri-urban settings.

## Figures and Tables

**Table 1:** Description of the three stands studied.

Stands	Altitude (m a.s.l.)	Slope (°)	Lithology ^1^	Area (ha)	pH
Urban forest (UF)	756	10	Sandstones and conglomerates	1.49	5.6
Peri-urban forest (PUF)	587	15	Feldspathic quartz sandstones	2.09	5.8
Natural forest (NF)	875	10	Sandstones and conglomerates	15.58	5.6

^1^ Geological map of Molise region, scale 1:50,000 (https://www.isprambiente.gov.it/Media/carg/405_CAMPOBASSO/Foglio.html) (accessed on 3 September 2024).

**Table 2:** The in-field and laboratory variables measured/calculated.

		Variables	Unit
Stand Structure	In-Field	Leaf area index (LAI: leaf area/ground area)	m^2^/m^2^
Leaf Functional Traits	In-Field	Fresh weight (FW)	g
		Leaf thickness (THICK)	mm
		Chlorophyll content (CHL: chlorophyll fluorescence ratio)	T940 nm/T660 mm
		Anthocyanin content (Anth: anthocyanin fluorescence ratio)	F660 nm/F525 nm
		Flavonol content (Flv: flavonols fluorescence ratio)	F660 nm/F325 nm
	Laboratory	Leaf area (LA)	cm^2^
		Dry weight (DW)	g
		Specific leaf area (SLA: leaf area/dry weight)	cm^2^/g
		Leaf dry matter content (LDMC: dry weight/fresh weight)	mg/g

**Table 3:** In the table, the following information are reported. *Satellite:* the name of the satellite used for the data collection. *Satellite data:* the parameter evaluated. Description: the satellite resolution and recording interval.

Satellite	Satellite Data	Description
Landsat 5/6/7/9	Land Surface Temperature (LST)	30 m of resolution—16 days
TerraClimate	Precipitation (PPT) [94]	4 km of resolution—monthly
	Soil Moisture (SMOI) [94]	
	Actual Evapotranspiration (ACTEVP) [94]	

## Data Availability

The authors will make the raw data supporting this article’s conclusions available upon request.

## Supplementary Materials

The following supporting information can be downloaded at: https://www.mdpi.com/article/10.3390/plants14020285/s1, Table S1: Phytosociological relevés of the sampling stands; Table S2: Climatic data of the three stands in the Campobasso municipality: natural forest (NF), peri-urban forest (PUF), and urban forest (UF). A–M: April–May 2023, J–J: June–July 2023, J–A: July–August 2023; Table S3: EIVE 1.0 indicator values for the niche position of L (light), T (temperature), M (moisture), R (reaction), and N (nitrogen availability) from Dengler et al. [34] for the 70 taxa listed in Table S1.
