# Supplementary material for: Quercus cerris Leaf Functional Traits to Assess Urban Forest Health Status for Expeditious Analysis in a Mediterranean European Context
Source: Plants (Basel). 2025 Jan 20;14(2):285. doi: 10.3390/plants14020285 (PMC11768225; doi:10.3390/plants14020285)
Supplement: Supplementary file 1 [file plants-14-00285-s001.zip › plants-3405137-supplementary.pdf]

plants

Supplementary materials

Quercus cerris PFTs analysis to assess urban forests health status for expeditious analysis in a Mediterranean Europe contest

Luca Quaranta [1,*], Piera Di Marzio [1,2] and Paola Fortini [1,2]

1 Department Bioscience and Territory, University of Molise, I-86090 Pesche (IS), Italy; l.quaranta1@studenti.unimol.it; piera.dimarzio@unimol.it; fortini@unimol.it
2 National Biodiversity Future Center (NBFC), 90133 Palermo, Italy
* Correspondence: l.quaranta1@studenti.unimol.it

Table S1. Phytosociological relevés of the sampling stands.

Slope (%)	10	15-18	10
Aspect	E	W/E	N/W
Elevation m a.s.l.	875	584	657
Soil surface aspect, rocky outcrops (%)	-	-	-
Soil surface aspect, stoniness (%)	-	-	5
Canopy layer cover (%)	70	90	80
Shrubs layer cover (%)	85	90	75
Herbaceous layer cover (%)	80	40	70
Forest stand acronym: NF natural forest; PUF peri-urban forest; UF urban forest	NF	PUF	UF
Total species	59	37	36
**Roso arvensis-Quercetum cerridis** Ubaldi 2003			
Quercus cerris L.	4	3	4
Rosa arvensis Huds.	1	1	1
Lonicera caprifolium L.	+	+	1
**Crataego laevigatae-Quercion cerridis** Arrigoni 1997			
Quercus frainetto Ten.	3	2	3
Ligustrum vulgare L.	2	2	3
Digitalis michranta Roth ex Schweigg.	+	.	.
Genista tinctoria L.	1	.	.
**Fagetalia sylvaticae** Pawłowski in Pawłowski, Sokołowski & Wallisch 1928			
Sorbus aucuparia L.	+	1	1
Allium pendulinum Ten.	+	+	1
Asperula taurina L. subsp. *taurina*	+	.	.
Geranium versicolor L.	1	.	.
Neottia nidus-avis (L.) Rich.	+	.	.
Scrophularia nodosa L.	+	.	.
Acer pseudoplatanus L.	1	.	.

**Carpino-Fagetea** Jakucs ex Passarge 1968			
Rubus hirtus Waldst. & Kit. group	2	1	1
Vicia sepium L.	2	1	+
Acer campestre L.	1	1	2
Melica uniflora Retz.	1	1	1
Clematis vitalba L.	1	1	1
Luzula forsteri (Sm.) DC.	+	+	+
Daphne laureola L.	1	+	.
Aremonia agrimonoides (L.) DC. subsp. *agrimonoides*	2	.	1
Viola reichenbachiana Jord. ex Boreau	1	.	+
Ulmus minor Mill.	.	2	+
Quercus petraea (Matt.) Liebl.	3	.	.
Corylus avellana L.	1	.	.
Fragaria vesca L. subsp. *vesca*	1	.	.
Ajuga reptans L.	1	.	.
Solidago virgaurea L.	+	.	.
Primula vulgaris Huds.	.	+	.
Prunus avium (L.) L.	.	+	.
Pulmonaria hirta L.	.	+	.
**Quercetalia pubescenti-petraeae** Klika 1933			
**Quercetea pubescentis** Doing-Kraft ex Scamoni et Passarge 1959			
Quercus pubescens Willd.	+	2	3
Lathyrus venetus (Mill.) Wohlf.	1	+	2
Fraxinus ornus L. subsp. *ornus*	1	+	.
Sorbus torminalis (L.) Crantz	+	.	2
Geum urbanum L.	+	+	+
Carpinus orientalis Mill.	2	.	.
Echinops sphaerocephalus L. subsp. *sphaerocephalus*	+	.	.
Scutellaria columnae All.	+	.	.
Sedum cepaea L.	+	.	.
Cornus mas L.	1	.	.
Acer opalus Mill. subsp. *obtusatum* (Waldst. & Kit. ex Willd.) Gams	+	.	.
Helleborus foetidus L. subsp. *foetidus*	.	+	.
Arum italicum Mill. subsp. *italicum*	.	+	.
**Wide ecology forest species**			
Brachypodium sylvaticum (Huds.) P.Beauv. subsp. *sylvaticum*	2	1	2
Ruscus aculeatus L.	2	1	1
Hedera helix L. subsp. *helix*	1	3	3
Dioscorea communis (L.) Caddick & Wilkin	+	1	.
Veronica officinalis L.	1	.	.
**Crataego-Prunetea** Tüxen 1962	·	·	·

Crataegus monogyna Jacq.	3	3	1
Prunus spinosa L. subsp. *spinosa*	1	1	2
Cornus sanguinea L. subsp. *hungarica* (Kárpáti) Soó	1	1	+
Emerus major Mill.	+	.	+
Rosa canina L.	1	.	.
Pyracantha coccinea M.Roem.	.	.	+
**Quercetea ilicis** Br.-Bl. in Br.-Bl., Roussine & Nègre 1952			
Asparagus acutifolius L.	+	1	.
Lamium album L. subsp. *album*	.	.	+
**Trifolio-Geranietea** Müller 1962			
Urtica dioica L.	+	+	+
Artemisia vulgaris L.	+	.	.
Astragalus glycyphyllos L.	+	.	.
Geranium columbinum L.	.	.	+
Salvia verbenaca L.	.	.	+
**Festuco-Brometea** Br.-Bl. & Tüxen ex Br.-Bl. 1949	·	·	·
Festuca circumediterranea Patzke	1	1	1
Carex flacca Schreb. subsp. *eriostachya*	1	1	1
Dactylis glomerata L.	1	.	1
**Molinio-Arrhenatheretea** Tüxen 1937			
Poa trivialis L.	1	+	1
Ranunculus bulbosus L.	1	.	.
Cruciata laevipes Opiz.	+	.	.
Carex pendula Huds.	.	+	.

Table S2. Climatic data for the three stands: natural forest (NF), peri-urban forest (PUF), and urban forest (UF) in the Campobasso municipality. A-M: April-May 2023, J-J: June-July 2023, J-A: July-August 2023.

STANDS	SMOI (mm)			LST (°C)			PPT (mm)			ACTEVP (mm)		
	A-M	J-J	J-A	A-M	J-J	J-A	A-M	J-J	J-A	A-M	J-J	J-A
NF	62.00	55.00	23.25	18.11	19.70	26.60	13.99	18.01	4.11	77.50	97.75	25.65
PUF	49.25	40.60	18.50	20.95	23.24	35.32	14.24	11.02	3.90	78.70	92.80	19.35
UF	48.15	40.20	18.25	22.18	24.84	33.00	14.40	11.67	3.92	80.55	93.15	19.85

Table S3. EIVE 1.0 indicator values for niche position of L (light), T (temperature), M (moisture), R (reaction), and N (nitrogen availability) from Dengler et al. [34] for the 70 taxa listed in Table S1.

Taxon	L	T	M	R	N
Acer campestre	5.0	5.0	4.2	6.8	5.8
Acer opalus subsp. *obtusatum*[1]	8.2	7.1	3.8	7.2	3.9
Acer pseudoplatanus	3.8	4.2	5.1	5.9	6.9
Ajuga reptans	5.3	4.1	5.0	5.6	5.7
Allium pendulinum	7.0	7.2	3.4	7.4	8.1
Aremonia agrimonoides subsp. *agrimonoides*	3.2	4.6	4.4	8.4	5.0
Artemisia vulgaris	7.5	4.5	4.1	6.3	7.6
Arum italicum subsp. *italicum*[2]	4.6	6.3	4.6	6.0	6.6
Asparagus acutifolius	6.0	6.8	2.0	6.0	4.4
Asperula taurina subsp. *taurina*	4.5	3.9	4.3	7.7	6.3
Astragalus glycyphyllos	5.8	4.4	3.8	6.8	3.7
Bituminaria bituminosa	8.2	6.7	2.8	7.3	4.3
Brachypodium sylvaticum subsp. *sylvaticum*	2.6	4.8	4.4	6.5	5.9
Carex flacca subsp. *eriostachya*[3]	6.7	4.3	5.1	7.7	3.4
Carex pendula	4.5	4.3	6.3	6.3	6.1
Carpinus orientalis	4.9	5.8	3.4	8.0	3.4
Clematis vitalba	6.3	4.8	4.4	7.1	6.4
Cornus mas	5.8	5.4	3.8	7.8	4.6
Cornus sanguinea subsp. *hungarica*	6.0	4.4	4.8	7.3	4.8
Corylus avellana	5.4	4.3	4.4	5.7	5.7
Crataegus monogyna	6.9	4.7	4.0	6.9	4.5
Cruciata laevipes	6.5	4.2	4.6	6.0	6.3
Dactylis glomerata	6.8	4.5	4.3	6.1	6.1
Daphne laureola	3.8	5.3	3.9	7.6	3.9
Digitalis micrantha[4]	8.2	4.6	4.2	8.8	5.3
Dioscorea communis	5.5	5.6	4.4	7.5	5.5
Echinops sphaerocephalus subsp. *sphaerocephalus*	8.2	5.8	2.6	6.6	6.9
Emerus major[5]	6.2	5.4	2.9	8.5	2.6
Festuca circummediterranea	9.6	4.0	1.6	7.0	2.2
Fragaria vesca subsp. *vesca*[6]	5.9	4.0	4.4	5.7	5.3
Fraxinus ornus subsp. *ornus*	6.3	5.9	3.2	8.1	4.1
Genista tinctoria	7.3	4.5	4.3	4.9	2.3
Geranium columbinum	7.4	4.9	3.3	6.8	5.2
Geranium versicolor	3.5	2.8	5.0	6.5	6.5
Geum urbanum	4.0	4.2	4.7	6.6	7.4
Hedera helix subsp. *helix*	4.0	7.5	3.7	5.7	5.1
Helleborus foetidus subsp. *foetidus*[7]	4.8	4.7	3.6	8.2	3.5
Lamium album subsp. *album*	6.9	3.9	4.3	6.3	9.8
Lathyrus venetus	3.4	5.5	4.1	5.9	4.8
Ligustrum vulgare	6.3	4.9	3.9	7.4	4.1

Lonicera caprifolium	5.9	5.3	4.2	7.4	4.3
Luzula forsteri	3.4	5.6	3.9	4.8	2.7
Melica uniflora	3.0	4.2	4.4	6.2	5.1
Neottia nidus-avis	1.5	4.1	4.5	7.1	5.2
Poa trivialis	6.0	4.1	5.7	5.9	6.9
Primula vulgaris[8]	5.3	4.7	4.5	6.6	5.1
Prunus avium	4.7	4.6	4.6	6.6	5.3
Prunus spinosa subsp. *spinosa*	7.4	4.4	3.4	5.9	5.2
Pulmonaria hirta	3.5	2.8	5.0	6.5	6.5
Pyracantha coccinea	7.1	5.9	3.9	6.2	4.0
Quercus cerris	6.1	5.7	3.6	5.5	4.4
Quercus frainetto	6.4	5.7	5.1	6.1	4.6
Quercus petraea	5.8	4.7	4.2	4.8	3.5
Quercus pubescens	6.5	6.0	3.2	7.2	3.9
Ranunculus bulbosus	7.8	4.6	3.4	6.7	3.1
Rosa arvensis	5.2	4.4	4.2	6.9	5.0
Rosa canina	7.2	4.3	3.9	6.4	4.3
Rubus hirtus aggr.	5.6	3.8	4.6	5.4	5.6
Ruscus aculeatus	4.2	6.3	4.0	6.6	4.4
Salvia verbenaca	8.0	5.9	2.7	6.9	3.9
Scrophularia nodosa	3.8	3.9	5.2	6.1	7.1
Scutellaria columnae	6.9	5.7	3.9	7.7	4.9
Sedum cepaea	4.6	6.1	3.5	5.1	5.1
Solidago virgaurea	5.0	3.7	4.3	4.4	4.2
Sorbus aucuparia	5.7	3.8	4.8	4.2	3.9
Sorbus torminalis	4.6	5.2	3.9	7.0	4.0
Ulmus minor	5.1	5.3	4.9	7.6	6.2
Urtica dioica	5.4	4.1	5.0	6.6	8.7
Veronica officinalis	5.0	3.6	4.0	3.4	3.5
Vicia sepium	5.5	3.9	4.4	6.3	5.4
Viola reichenbachiana	3.4	4.2	4.5	6.4	5.5

[1]In EIVE 1.0: *Acer obtusatum*; [2]In EIVE 1.0: *Arum italicum*; [3]In EIVE 1.0: *Carex flacca* ; [4]In EIVE 1.0: *Digitalis lutea* subsp. *australis*; [5]In EIVE 1.0: *Hippocrepis emerus*; [6]In EIVE 1.0: *Fragaria vesca*; [7]In EIVE 1.0: *Helleborus foetidus*; [8]In EIVE 1.0: *Primula acaulis*
